# Hand-Book of the Diagnosis and Treatment of Skin Diseases

**Published:** 1889-05

**Authors:** 


**Hand-Book of the Diagnosis and Treatment of Skin Diseases.** By ARTHUR VAN HARLINGEN, M. D., Professor of Diseases of the Skin in the Philadelphia Polyclinic and College for Graduates in Medicine; Clinical Lecturer on Dermatology in the Jefferson Medical College. Second edition. Enlarged and revised. With eight full-page plates, and other illustrations. 12mo, pp. xv., 410. Philadelphia: P. Blakiston, Son & Co., 1012 Walnut street. 1889. Buffalo: Peter Paul & Bro. Price, $2.50.

In this second edition the author has very much improved his work. It is better adapted to the wants of the general practitioner than any treatise on this subject with which we are familiar. Much new material has been added to the new edition, and the whole has been carefully revised. The arrangement of the diseases in alphabetical order is a good plan for a hand-book, and makes it of easy reference, without the aid of an index. Electricity, which is attracting so much interest in all departments of medicine just now, receives a due share of attention here, and clear suggestions as to its therapeutic applicability in various diseases of the skin are given.

